# Combined Exoscopic and Endoscopic Technique for Craniofacial Resection

**DOI:** 10.3390/curroncol28050336

**Published:** 2021-10-04

**Authors:** Kenichiro Iwami, Tadashi Watanabe, Koji Osuka, Tetsuya Ogawa, Shigeru Miyachi, Yasushi Fujimoto

**Affiliations:** 1 Department of Neurosurgery, Aichi Medical University, Nagakute 480-1195, Aichi, Japan; watanabe.tadashi.598@mail.aichi-med-u.ac.jp (T.W.); oosuka.kouji.973@mail.aichi-med-u.ac.jp (K.O.); miyachi.shigeru.752@mail.aichi-med-u.ac.jp (S.M.); 2 Skull Base Surgery Center, Aichi Medical University Hospital, Nagakute 480-1195, Aichi, Japan; yasushif@aichi-med-u.ac.jp; 3 Department of Otorhinolaryngology, Aichi Medical University, Nagakute 480-1195, Aichi, Japan; ogawa.tetsuya.304@mail.aichi-med-u.ac.jp

**Keywords:** craniofacial resection, endoscope, exoscope, transcranial approach, skull base

## Abstract

We determined the feasibility of the combined exoscopic-endoscopic technique (CEE) as an alternative to the microscope in craniofacial resection (CFR). This retrospective study was conducted at a single institution and included eight consecutive patients with head and neck tumors who underwent CFR between September 2019 and July 2021. During the transcranial approach, microsurgery was performed using an exoscope in the same manner as in traditional microscopic surgery, and an endoscope was used at the blind spot of the exoscope. The exoscope provided images of sufficient quality to perform microsurgery, while the sphenoid sinus lumen was the blind spot of the exoscope during anterior (*n* = 3) and anterolateral CFR (*n* = 2), and the medial aspect of the temporal bone was the blind spot of the exoscope during temporal bone resection (*n* = 2). These blind spots were visualized by the endoscope to facilitate accurate transection of the skull base. The advantages of the exoscope and endoscope include compact size, ergonomics, surgical field accessibility, and equal visual experience for neurosurgeons and head and neck surgeons, which enabled simultaneous transcranial and transfacial surgical procedures. All the surgeries were successful without any relevant complications. CEE is effective in transcranial skull base surgery, especially CFR involving simultaneous surgical procedures.

## 1. Introduction

Malignant head and neck tumors rarely arise from the nasal cavity, paranasal cavity, orbit, and hearing organs [1,2]. Among the many therapeutic approaches proposed for malignant head and neck tumors, complete surgical resection followed by postoperative radiotherapy reportedly provides the best outcomes [3,4,5]. Since the nasal cavity, paranasal cavity, orbit, and hearing organs are adjacent to the skull base, this region is often invaded by locally advanced malignant head and neck tumors.

**Figure 1:**
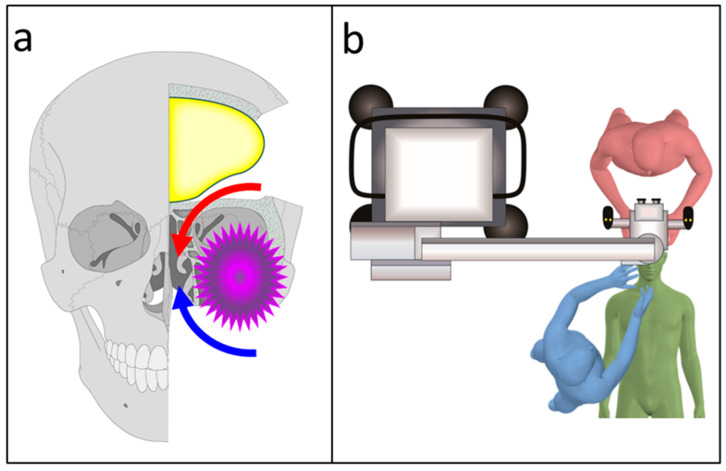
Schematic diagrams of craniofacial resection (CFR). (**a**) CFR combines both the transcranial (red) and transfacial (blue) approaches. (**b**) the transfacial approach is performed by head and neck surgeons (blue), whereas the transcranial approach is performed by neurosurgeons (red). Before 2019, a microscope was used during the transcranial approach of CFR. Green: patient.

At our institution, patients with malignant head and neck tumors, or benign aggressive lesions invading the skull base, are treated with craniofacial resection (CFR) [6,7]. CFR combines both the transcranial and transfacial approaches (Figure 1a): the transfacial approach is performed by head and neck surgeons, whereas the transcranial approach is performed by neurosurgeons. In CFR, a transcranial approach is critical in skull base osteotomy to permit the lesion to remain covered in the safety margin at the skull base (Figure 1a red arrow).

Although neurosurgeons are generally not very familiar with the management of malignant head and neck tumors, the efficacy and safety of surgical resection are expected to improve with the proactive involvement of neurosurgeons in the treatment of these tumors; hence, neurosurgeons should be a part of the multidisciplinary team [8]. CFR requires exceptional surgical skills and anatomical knowledge since the critical neural and vascular structures are within or adjacent to the skull base. An optimal view of the surgical field is required for the precise resection of the tumor at the appropriate site. CFR procedures are classified into anterior, lateral, anterolateral, and temporal bone resection (TBR), according to the position of the skull base resection [9].

**Figure 2:**
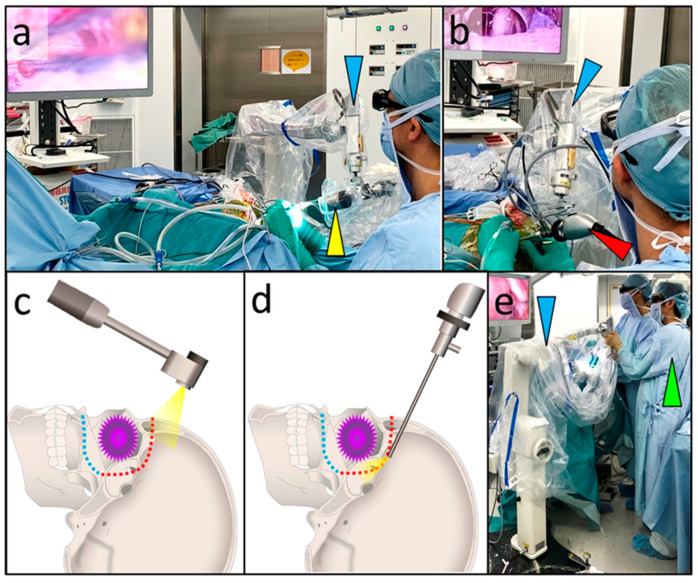
Photographs and schematic diagrams of combined exoscope and endoscope technique in the transcranial approach for craniofacial resection. (**a**) The majority of the transcranial procedures were performed using the VITOM-3D exoscope (yellow arrowhead), held by the Mitaka UniARM (blue arrowhead). The VITOM-3D does not have an eyepiece, and the surgeon performs the operation while viewing images (with 3D glasses) of the surgical field on a monitor. (**b**) Transections in the blind spots of the exoscope are visualized with an endoscope (red arrowhead), which is also held by the Mitaka UniARM (blue arrowhead). (**c**) Transcranial procedures are performed using VITOM-3D-delivered stereoscopic vision (with the exception of blind spots) in the same manner as traditional microsurgery. Purple: tumor. Red broken line: transcranial skull base transection. Blue broken line: transfacial skull base transection line. (**d**) The endoscope enables direct visualization behind the lesions, a location that is difficult to access with an exoscope or microscope. Purple: tumor. Red broken line: transcranial skull base transection. Blue broken line: transfacial skull base transection line. (**e**) The exoscope and endoscope held by the Mitaka UniARM (blue arrowhead) are controlled by scopists (green arrowhead); 3D, three-dimensional.

Before 2019, microscopes were used during the transcranial approach in CFR (Figure 1b) [6,9]. The visualization tools used in skull base surgery should permit magnification, illumination, and favorable ergonomics. Owing to the advances in digital imaging, the three-dimensional (3D) exoscope has been increasingly used as an alternative to microscopes in cranial surgery. In the last decade, several types of exoscopes have been developed and adapted for use in various neurosurgical procedures [10,11,12,13]. An exoscope usually does not have an eyepiece, and surgeons operate while viewing images (with 3D glasses) of the surgical field on a monitor. The VITOM-3D exoscopic system (Karl Storz GmbH, Tuttlingen, Germany) is less expensive and smaller than microscopes and possesses the additional advantage of better image quality, ergonomics, and a small footprint [14,15,16,17]. Previous studies have rated the illumination and magnification of the VITOM-3D in the deep surgical field as inferior compared to those of microscopes [16,18]. To compensate for this shortcoming of VITOM-3D, we used an endoscope to visualize the tumor and anatomical landmarks around corners and in exoscope blind spots. An endoscope provides better illumination and visualization in deep and narrow surgical fields. Furthermore, angled scopes provide better visualization of corners and hidden areas; this allows skull base transection without a direct line of sight. We have utilized this combined exoscopic and endoscopic technique (CEE) for all transcranial skull base surgeries since 2019 (Figure 2a,b) [19]; among these, eight cases were of CFR.

In this study, we report successful CFR with CEE in the transcranial approach. CEE provided improved ergonomics, visualization, and illumination compared with the conventional microscope. The introduction of digital visualization devices, such as exoscopes, is expected to result in major advancements in skull base surgery [20], and our experience would be beneficial for surgeons treating malignant head and neck tumors.

## 2. Materials and Methods

### 2.1. Patients

This is a retrospective case series of eight consecutive patients who underwent CFR at the Aichi Medical University Hospital between September 2019 and July 2021. Computed tomography (CT) and magnetic resonance imaging were performed preoperatively to evaluate the anatomy of the skull base and the lesions, as well as postoperatively (at 0.5- or 1-mm-thick tissue slices) to evaluate the surgical results. This study was approved by the ethics review committee of Aichi Medical University Hospital (Approval Number: 2020-020), and written informed consent was obtained from all patients. Information on the following parameters was collected for data analysis: (1) patient demographics (age, sex, and tumor histopathology), (2) operation indices (type of surgery, total operation time, neurosurgery operation time [from the start of the craniotomy until completion of the skull base osteotomy]), and (3) postoperative course and complications. Complications were thoroughly assessed through medical examinations and face-to-face interviews during the follow-up period and categorized into three groups: central nervous system complications (intracranial hemorrhage, cerebral infarction, cerebrospinal fluid leakage, and meningitis), wound complications (surgical site infection and skin flap necrosis), and systemic complications (pneumonia and sepsis).

### 2.2. Surgical Techniques Used in the Transcranial Approach

All patients underwent CFR, which combined transcranial and transfacial approaches. The transcranial approach was performed with the combined use of an exoscope and endoscope. Scalp incisions, craniotomy, most skull base transections, and wound closures were performed using a 3D exoscope (VITOM 3D) (Figure 2a,c). The areas of the transection that were in the blind spots of the exoscope were transected with the use of a rigid 0° or 30° endoscope (outside diameter of 4 mm and length of 18 cm) (Karl Storz, Tuttlingen, Germany) (Figure 2b,d). The exoscope and endoscope were fixed to a pneumatic articulated arm (Mitaka UniARM, Mitaka Kohki Co, Tokyo, Japan), which was controlled by a second doctor (scopist, Figure 2a,b,e). 

### 2.3. Treatment Strategy for the Head and Neck Malignant Tumor

The surgical indication for CFR was a tumor involving the anterior and/or lateral skull base, with the possibility of an en bloc resection. To ensure the absence of residual tumor, the osteotomy was planned to extend at least 5 mm beyond the surgical margin established during the preoperative simulation [6,21]. Using the navigation system, we cut the skull base according to the preoperative plan. The contraindications to surgical management of malignant head and neck tumors are usually related to the difficulty in achieving en bloc resection with tumor-free margins. In our patients, these contraindications included distant metastasis and invasion of the sphenoid sinus, cavernous sinus, clivus, internal carotid artery, and sigmoid sinus [6,7].

The surgical strategy for head and neck tumors has been previously described [6,7,8,21,22,23]. Briefly, the head and neck surgeons performed facial dissection or endoscopic endonasal dissection. Surgical neck dissection was also performed in patients with cervical lymph node metastases by head and neck surgeons, followed by cranial dissection performed by neurosurgeons. In both facial and cranial dissections, osteotomy was performed using a high-speed drill with a 3-mm round burr or bone chisel. The tumor was then resected in an en bloc fashion. Following resection of the tumor, the dura mater defect was closed using a nonvascularized fascia graft. The plastic surgeons reconstructed the cranial base defect using a galeal flap, rectus abdominis myocutaneous free flap, or omental free flap. After CFR, histopathological examination of the surgical margin was performed in all cases.

Based on the tumor histology and margin status, postoperative radiotherapy was advocated. Adjuvant radiotherapy was initiated within 8 weeks postoperatively, with a planned dose of 60 Gy (administered as five 2-Gy fractions per week).

## 3. Results

Clinical and demographic characteristics for the enrolled patients are summarized in Table 1.

### 3.1. Patient Characteristics

In total, eight patients (men: *n* = 5) were included. The patients’ mean (±standard deviation [SD]) age at the time of surgery was 51.8 ± 23.8 years. Of the eight patients, three (37.5%) had previously received treatment before surgery. The histological diagnosis was squamous cell carcinoma in three patients (37.5%), neuroblastoma in two patients (25%), adenocarcinoma in one patient (12.5%), malignant peripheral nerve sheath tumor in one patient (12.5%), and aneurysmal bone cyst in one patient (12.5%). 

### 3.2. Intraoperative Findings

**Figure 3:**
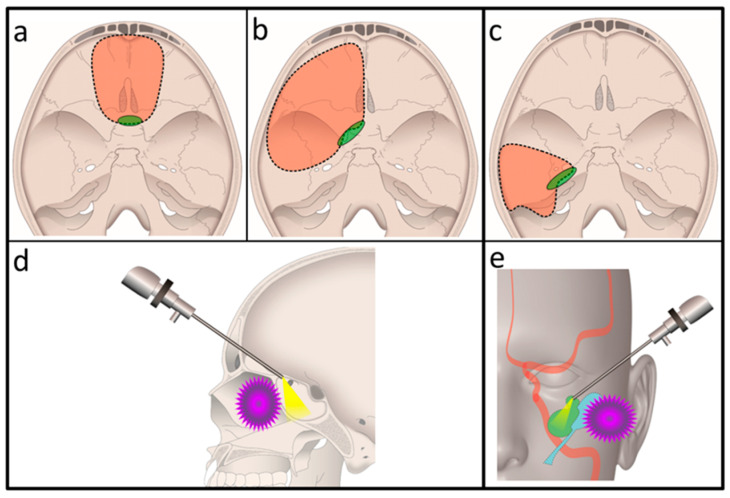
Schematic diagram of skull base site treated with craniofacial resection. (**a**) Anterior craniofacial resection. (**b**) Anterolateral craniofacial resection. (**c**) Temporal bone resection. Red region enclosed by dotted line: skull base resection region. Green ellipses: the points at which an endoscope was used. (**d**,**e**) Transcranial endoscope use. (**d**) Anterior and anterolateral CFR. An endoscope is inserted into the sphenoid sinus through the opening created by the transcranial sphenoidotomy. The anterior wall and floor of the sphenoid sinus are transected under endoscopic visualization. Purple: tumor. (**e**) Temporal bone resection. The medial segment of the temporal bone is transected under direct visualization of the important anatomical structures using an endoscope. Red: internal carotid artery. Green: inner ear. Sky blue: middle ear and eustachian tube. Purple: tumor CFR, craniofacial resection.

We performed anterior CFR (Figure 3a), anterolateral CFR (Figure 3b), and temporal bone resection (Figure 3c) in 3, 3, and 2 patients, respectively. 

### 3.3. Operation Time and Intraoperative Blood Loss

The mean (±SD) total operation time was 811.5 ± 385.1 min, and the mean (±SD) neurosurgery operation time was 200.0 ± 56.8 min. The mean (±SD) amount of intraoperative blood loss was 698.4 ± 512.2 mL. Compared to conventional surgery using a microscope, no delays or interruptions owing to poor visibility in the surgical field were observed during transcranial surgical procedures [7,23,24]. However, due to the very small number of cases and wide variety of surgery types, it was not possible to objectively evaluate the operation times and amount of bleeding.

### 3.4. Combined Exoscopic and Endoscopic Technique in the Transcranial Approach

The exoscope provided excellent 3D images of the transcranial surgical field in all eight cases. The exoscopic portion of the transcranial approach was performed according to the same steps as the traditional CFR, which utilized a microscope. As the surgical view provided by the exoscope became obscured in the deep and narrow surgical field during skull base transection in all eight cases, we substituted it with an endoscope to obtain a better surgical view. Green ellipses in Figure 3a–c show the points at which we used an endoscope. 

The transection of the bony wall of the sphenoid sinus (i.e., transcranial sphenoidotomy) was required for the anterior and anterolateral CFR, and this was performed with an exoscope as previously described (Figure 3a,b, green ellipses) [6] We inserted an endoscope into the sphenoid sinus through an opening created by the transcranial sphenoidotomy (Figure 3d) and transected the anterior wall and floor of the sphenoid sinus under clear visualization. For temporal bone resection, preserving important structures, such as the internal carotid artery and jugular bulb located on the medial side of the temporal bone, was critical; however, observing these vital anatomical structures under an exoscope was challenging since the middle ear and external ear involved in the tumor obstructed the field of view (Figure 3c, green ellipse). Therefore, an endoscope was inserted into the extradural space of the lateral skull base, and the medial segment was transected under direct visualization (Figure 3e).

**Figure 4:**
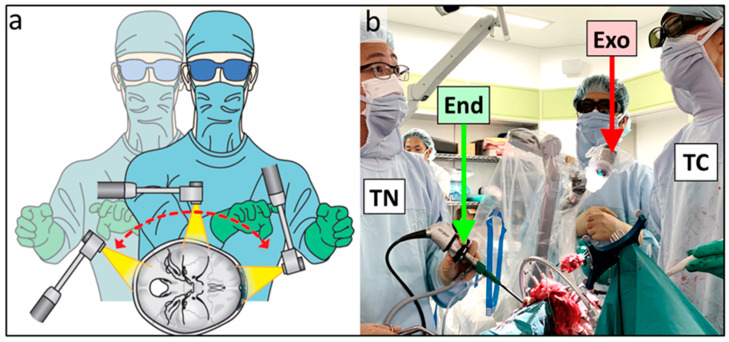
(**a**) Schematic diagram of the surgeon’s posture while using an exoscope. By adjusting the angle of the exoscope and the surgeon’s arms, transcranial procedures can be performed in a comfortable posture, regardless of the angle of the visual axis and patient positioning. (**b**) Intraoperative photograph showing two surgeons simultaneously performing transcranial and transnasal procedures during anterior craniofacial resection. TN: a surgeon performing a transnasal procedure, TC: a surgeon performing a transcranial procedure, End: endoscope, Exo: exoscope.

**Figure 5:**
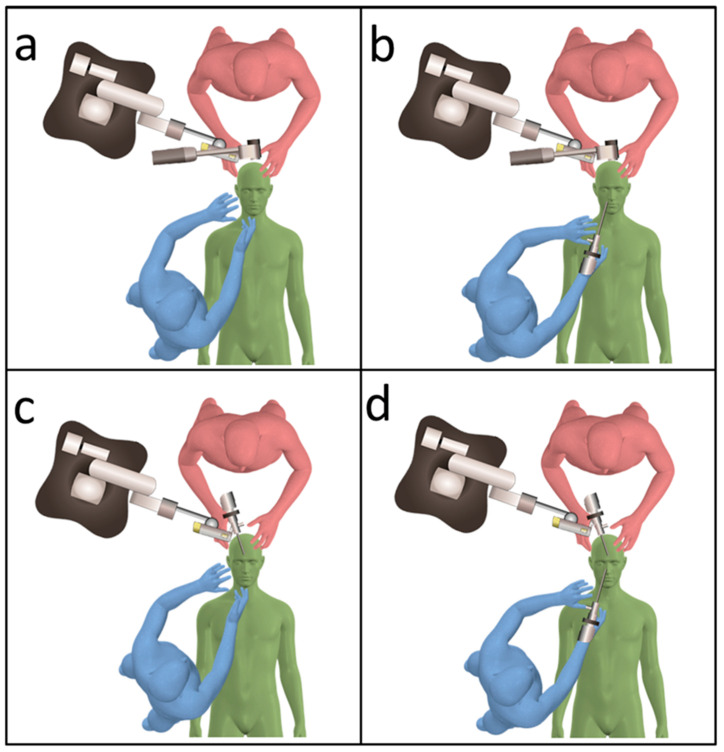
Schematic diagram of the two-surgeon set-up utilized in this case series. (**a**) Transcranial surgeon (red) using an exoscope and transfacial surgeon (blue) using no observation device. (**b**) Transcranial surgeon (red) using an exoscope and transnasal surgeon (blue) using an endoscope. (**c**) Transcranial surgeon (red) using an endoscope and transfacial surgeon using (blue) no observation device. (**d**) Transcranial surgeon (red) and transnasal surgeon (blue) both using endoscopes. For any combination, neither the exoscope nor endoscope interfered with the field of vision or procedures carried out by either surgeon. Green, patient.

**Figure 6:**
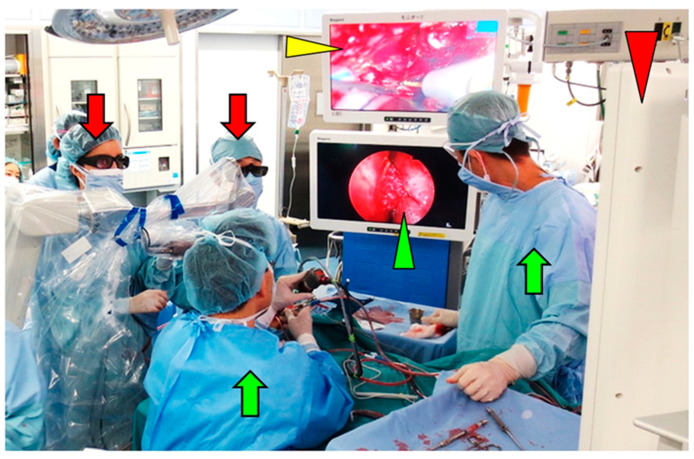
Intraoperative photograph showing two teams simultaneously performing transcranial and transnasal procedures during anterior craniofacial resection. Red arrows: transcranial team, green arrows: transnasal team, red arrowhead: display monitor for the transcranial team, green arrowhead: intranasal surgical field on the display monitor for the transnasal team, yellow arrowhead: intracranial surgical field on the display monitor for the transnasal team.

Besides adequate optics, working environment ergonomics is also an important factor for achieving good clinical outcomes during CFR, which requires the coordination of multiple surgeons and the use of a range of surgical devices. We were able to perform microsurgery using an exoscope while assuming a comfortable posture (Figure 1a,e); this was regardless of the angle of the visual axis and patient positioning (Figure 4a). In all the eight surgeries, there were times when the transcranial and transfacial surgeons were simultaneously performing procedures (Figure 4b). There were four combinations of observation devices used by the two teams simultaneously (Figure 5, Table 1); in any combination, neither the exoscope nor endoscope interfered with the field of vision or the procedures carried out by either surgeon. Furthermore, the combined use of the endoscope and exoscope enabled all the members of the multidisciplinary team to share the same image of the surgical field (Figure 6). We did not experience any technical problems with the exoscope or endoscope in all eight surgeries.

### 3.5. Postoperative Findings

Histologically, tumor-free margins were obtained in seven patients (87.5%), while postoperative histopathology results showed tumor cells at the resected margin in one patient (12.5%) Three patients (37.5%) received postoperative radiotherapy, while no central nervous system complication and perioperative mortality were observed. Minor surgical site infection and postoperative sepsis were detected in one patient (12.5%) who was successfully treated with antibiotic therapy. There were no other wound or systemic complications. The mean (±SD) intensive care unit length of stay (LOS) and total hospital LOS were 1.75 ± 0.70 and 26.8 ± 20.1 days, respectively. The mean (± SD) follow-up duration was 9.5 ± 7.4 months. During the follow-up period, one patient (12.5%) experienced local tumor recurrence.

### 3.6. Typical Cases

#### 3.6.1. Case 1

**Figure 7:**
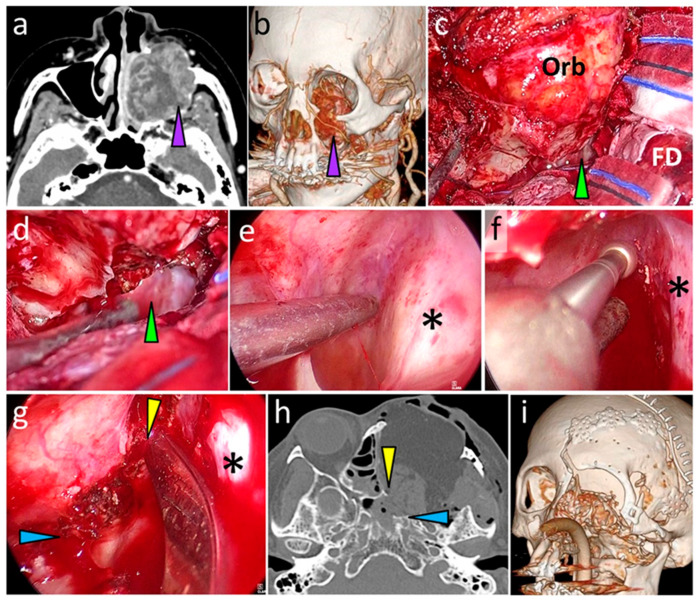
CT images and photographs of Case 1. (**a**,**b**) Preoperative axial CT and 3D-CT images showing maxillary sinus carcinoma invading the orbital apex and root of the pterygoid process (purple arrowheads). (**c**,**d**) Intraoperative exoscopic photographs showing the opened sphenoid sinus (green arrowheads). Orb: orbit. FD: frontal dura. (**e**–**g**) Intraoperative endoscopic photographs showing the sphenoid sinus lumen. *: midline intersphenoid septum. (**e**) Wide and clear visualization of the sphenoid sinus lumen. (**f**,**g**) The anterior wall (yellow arrowhead) and floor (blue arrowhead) of the sphenoid sinus are transected using a drill and curved chisel. (**h**) Postoperative axial CT scan showing the cut end of the anterior wall (yellow arrowhead) and floor (blue arrowhead) of the sphenoid sinus. (**i**) Postoperative 3D-CT image. CT, computed tomography; 3D, three-dimensional.

A 79-year-old woman with maxillary sinus squamous cell carcinoma involving the orbital apex and root of the pterygoid process (Figure 7a,b) underwent anterolateral CFR (Figure 3b). Following the transfacial dissection by the head and neck surgeons, transcranial dissection was performed. Using an exoscope, a frontotemporal craniotomy and superolateral sphenoidotomy [6] were conducted (Figure 7c, green arrowhead). Although it was difficult to observe the entire lumen of the sphenoid sinus using the exoscope following superolateral sphenoidotomy, this surgical procedure provided an adequate opening for the insertion of the endoscope into the sphenoid sinus (Figure 7d, green arrowhead). By using an endoscope (as shown in Figure 3d), clear visualization of the midline intersphenoid septum and anterior wall and floor of the sphenoid sinus (Figure 7e) was possible. The anterior wall and floor were subsequently transected at the planned position by using a high-speed drill and curved chisel (Figure 7f–h). Figure 7i shows the skull base resection range in a postoperative 3D-CT image.

#### 3.6.2. Case 2

**Figure 8:**
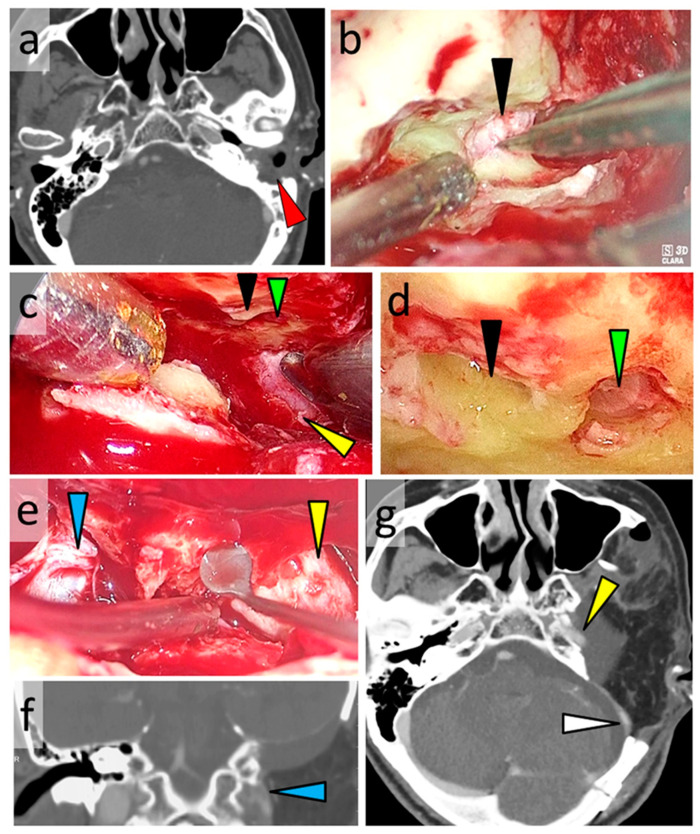
CT images and photographs of Case 2. Subtotal temporal bone resection performed in a patient with external auditory canal squamous cell carcinoma. (**a**) Preoperative axial CT image showing left external auditory canal carcinoma involving the temporomandibular joint, tympanic cavity, and mastoid air cells. (**b**) Intraoperative exoscopic photographs showing the opened internal auditory canal (black arrowhead). (**c**–**e**) Intraoperative endoscopic photographs. (**c**) The carotid canal is opened following the transection of the internal auditory canal (black arrowhead) and cochlea (green arrowhead). Yellow arrowhead: the internal carotid artery. (**d**) The 30° angled endoscopic photograph showing the cut end of the internal auditory canal (black arrowhead) and cochlea (green arrowhead). (**e**) The lateral wall of the carotid canal (yellow arrowhead) and jugular foramen (blue arrowhead) are carefully dissected. (**f**) Postoperative coronal CT image. Blue arrowhead: opened jugular foramen. (**g**) Postoperative axial CT image. Yellow arrowhead: opened carotid canal. White arrowhead: sigmoid sinus. CT, computed tomography.

A 68-year-old man with external auditory canal squamous cell carcinoma involving the temporomandibular joint had undergone subtotal temporal bone resection (Figure 3c). CT revealed tumor invasion into the tympanic cavity and mastoid air cells (Figure 8a). Following transfacial dissection by the head and neck surgeons, transcranial dissection was performed. Using an exoscope, a right temporo-suboccipital craniotomy was performed, and the temporal lobe and dura mater were subsequently elevated from the lateral skull base bone. The internal auditory canal and cochlea were opened, without exposing the middle ear (Figure 8b). After transection of the internal auditory canal and cochlea using the exoscope, an endoscope was inserted into the extradural space of the lateral skull base (Figure 3c,d). With the assistance of the endoscope, the carotid canal was opened without exposing the eustachian tube (Figure 8c). Figure 8d shows an endoscopic view of the cut end of the internal auditory canal and cochlea. Using an endoscope, the lateral wall of the carotid canal and jugular foramen was carefully dissected (Figure 8e). En bloc resection of the tumor was achieved by performing extradural resection of all sites other than the internal auditory canal (Figure 8f,g).

### 3.7. The Other Cases

**Figure 9:**
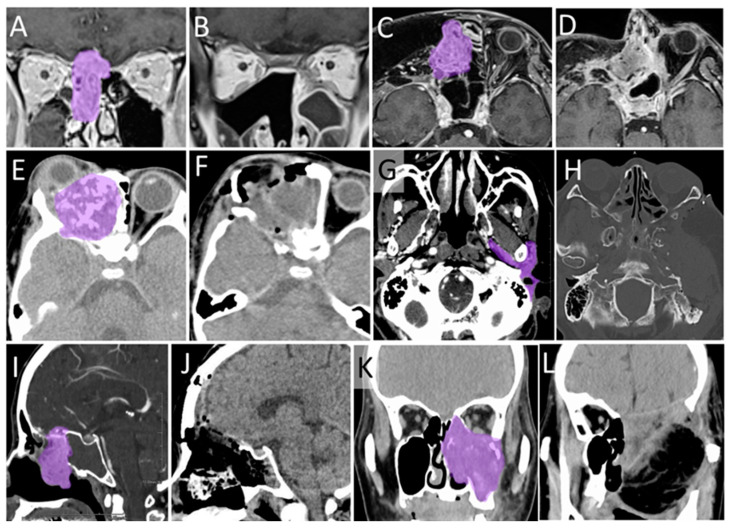
Pre- and postoperative images of Cases 3–8. (**A**,**B**) Gadolinium-enhanced T1 weighted coronal MRI images of Case 3, (**C**,**D**) Gadolinium-enhanced T1 weighted MRI images of Case 4, (**E**,**F**) CT images of Case 5, (**G**,**H**) CT images of Case 6, (**I**,**J**) Sagittal CT images of Case 7, (**K**,**L**) Coronal CT images of Case 8. (**A**,**C**,**E**,**G**,**I**,**K**) Preoperative images, (**B**,**D**,**F**,**H**,**J**,**L**) postoperative images. Purple; tumor. MRI, magnetic resonance imaging; CT, computed tomography.

Figure 9 shows the pre- and postoperative images of Cases 3–8.

## 4. Discussion

The exoscope is a recent technological innovation that has been used as an alternative to the microscope in neurosurgery and other surgical fields [16,25,26,27]. The main advantages of the VITOM-3D exoscope include its ability to be conveniently and rapidly interchanged with an endoscope [20], improved ergonomics, lower costs, ease of portability, as well as educational benefits owing to the use of a shared operative view. The main drawbacks of the VITOM-3D exoscope are the limited illumination and pixilation at high magnifications when utilizing deep and narrow surgical corridors [16,18]. Nevertheless, these drawbacks can be overcome by using an endoscope. The additional use of an endoscope provides a superior and wide-field view of deep and narrow surgical fields, since it possesses a light source at its tip, and can be used with a range of angled lenses. While a previous study reported difficulties with VITOM-3D repositioning [15], this issue was mitigated in our hospital by utilizing a pneumatic scope holder and the assistance of a scopist [19]. We believe that CEE can satisfy the need for 3D stereopsis, illumination, and magnification in the deep surgical field, and facilitate the complete resection of skull base tumors. 

The effective time for transcranial endoscope use was limited to the end stage of the skull base transection; nonetheless, it was very effective and provided a wide and clear view of the sphenoid sinus lumen and medial aspect of the temporal bone, which were without direct line of sight. The visualization of these regions had been difficult with the use of microscopes in the traditional CFR approach; thus, in the past, transections were performed with a curved chisel, without direct visualization. In our case series, these regions were safely and accurately transected using a drill and curved chisel under direct visualization with an endoscope. 

As shown in Figure 1a, the microscope is large and overhangs the patient’s head. Therefore, when a neurosurgeon and head and neck surgeon simultaneously perform surgical procedures, it is necessary to devise the patient’s position, the operators’ position, and the arrangement of the other surgical equipment. Moreover, the small camera head of the exoscope and endoscope do not interfere with the surgeon’s access to the surgical field during simultaneous transcranial and transfacial procedures. The small footprint of the exoscope and endoscope provided ample space for the members of the multidisciplinary team to access the surgical field and position other surgical equipment. Besides facilitating optimal working environment ergonomics, both the exoscope and endoscope enabled all members of the multidisciplinary team to share the same image of the surgical field. 

The mean operation times (total, 811.5 ± 385.1 min; neurosurgery 200.0 ± 56.8 min) and the mean amount of intraoperative blood loss (698.4 ± 512.2 mL) were comparable to those observed in our previous reports on CFR using a microscope (total operation time for TBR, 765.8 ± 139.8 min [28]; neurosurgery operation time for TBR, 161.2 ± 68.5 min [28]; intraoperative blood loss for TBR, 1627.0 ± 1568.7 mL [28]; total operation time for anterolateral CFR, 942 (range, 616–1945) min [7]; and intraoperative blood loss for anterolateral CFR, 1426 (range, 500–6228) ml [7]). Considering the ergonomics and small footprint of the exoscope and endoscope, and the reduction of blind spots by the endoscope, we assume CEE to be better than a microscope for a transcranial approach in CFR.

This technical note only focused on our preliminary experience with the CEE in the transcranial approach for CFR. Considering the transfacial approach, to date, only an endoscope has been used for transnasal procedures in anterior CFR at our hospital. However, we believe that an exoscope could be useful for precise surgical procedures, such as facial nerve dissection during the transfacial approach for CFR. Considering the many benefits of the CEE, it possesses the potential to become a valuable tool in the armamentarium to not only neurosurgeons, but also head and neck surgeons involved in the treatment of skull base tumors.

### Limitations

The main limitation of this report was the use of a single-center design, and the lack of quantifiable outcome measures; thus, our findings are anecdotal and have limited generalizability. The small number of cases and the short follow-up period are also major limitations. Nevertheless, with an increasing number of cases treated at our hospital, and extended postsurgical observation times, we would be able to gain additional insight into the long-term success rate of the CEE for CFR; moreover, the incidence of tumor recurrence and surgical complications should also be considered. 

## 5. Conclusions

This report described our preliminary experience with the CEE in the transcranial approach for CFR. The combined use of an exoscope and endoscope enabled a clear and comprehensive surgical field of view, and skull base transection under direct visualization. It also facilitated optimal working environment ergonomics. Our experience suggests that the CEE is a useful option for the transcranial approach to CFR. Since malignant head and neck tumors are rare, our preliminary experiences presented in this report can be used to guide surgeons who seldom encounter this condition in their practice.

## Figures and Tables

**Table 1:** Clinical data of eight patients who underwent CFR using exoscope and endoscope combination technique.

Case No.	Type of CFR	Approach/ Observation Device		Operation Time (min)		Bleeding (mL)	Complication	PORT	FU (mo)
		NS	HNS	Total	NS				
1	Anterolateral	FT/ Exo + End	TF/ None	1095	229	1670	SSI, Sepsis	No	14
2	TBR	TS/ Exo + End	TF/ None	925	284	437	None	No	8
3	Anterior	BF/ Exo + End	TN/ End	430	160	230	None	Yes	24
4	Anterolateral	FT/ Exo + End	TF/ None	1138	244	1030	None	Yes	14
5	Anterior	BF/ Exo + End	TN/ End	360	95	89	None	No	7
6	TBR	Temporal/ Exo + End	TF/ None	1010	192	996	None	No	4
7	Anterior	BF/ Exo + End	TN/ End	295	203	550	None	Yes	3
8	Anterolateral	FT/ Exo + End	TF/ None	1239	193	585	None	No	2

CFR, craniofacial resection; NS, neurosurgeon; HNS, head and neck surgeon; FT, frontotemporal; TS, temporosuboccipital; BF, bifrontal; Exo, exoscope; End, endoscope; TF, transfacial; TN, transnasal; SSI, surgical site infection; PORT, postoperative radiotherapy; FU, follow up.

## Data Availability

The data that support the findings of this study are available from the corresponding author, KI, upon reasonable request.
